# Biomaterials and Agents: Pharmaceutical and Biomedical Applications in Dental Research

**DOI:** 10.3390/pharmaceutics16070894

**Published:** 2024-07-04

**Authors:** Pavel Seredin, Dmitry Goloshchapov, Tatiana Litvinova

**Affiliations:** 1 Department of Solid-State Physics and Nanostructures, Voronezh State University, 394018 Voronezh, Russiacentr_rus_yaz@mail.ru (T.L.); 2 Psycholinguistic Textual Modelling Laboratory, Voronezh State Pedagogical University, 394043 Voronezh, Russia

Diseases of the oral cavity are of great importance due to the influence of dental status on a person’s social level [1,2]. In recent years, the global healthcare system has been faced with a number of challenges, including caries and periodontal diseases, which are the most common human diseases in the world [3]. It is safe to say that these diseases present a serious health problem in most countries of the world [3], a fact that is becoming evident in connection with the steady increase in the cost of treating oral diseases [4], as well as the evidence of the relationship between complicated caries or periodontitis and a number of common diseases [5,6,7,8].

That is why the focus of a lot of studies is on new biomaterials [9] and pharmaceutical agents for the treatment [10], prevention [11], mineralization [12], regeneration, and restoration of dental tissue [13,14], as well as the suppression of the microbial activity of pathogenic bacteria in the oral cavity [15,16].

This Special Issue, entitled **“Biomaterials and Agents: Pharmaceutical and Biomedical Applications in Dental Research”**, addresses the issues and challenges associated with the development, use, and modification of new biomaterials and agents for dental applications, and the study of their effects on mineralized tissues of the human body and processes related with them.

The research objects and scientific developments that this Special Issue is devoted to are critical technologies and breakthroughs in regenerative medicine and materials science, bioactive materials, and pharmaceutical agents.

Our Special Issue consists of 33 papers (26 scientific articles and 7 reviews) selected after a rigorous peer review process. 

Contributions were made by various countries and entire regions, as well as international groups of scientists (Russia and Australia, Belgium and Japan, India and Spain, Germany and China). 

The distribution of the articles by the authors’ countries is shown in Figure 1.

Most of the contributions (articles and reviews) provide an insight into the latest cutting-edge technologies and discuss the development and testing of new nanobiomaterials with a promising potential for tissue engineering (restoration and regeneration of hard tissues), assessment of mineralization potential, development of new peptides for dentistry and orthopedics applications, evaluation of antibacterial and antibiofilm effects, as well as drug delivery and targeting.

To help our readers gain more insights into this Special Issue’s content, we applied some basic natural language processing (NLP) techniques to the analysis of the article texts and made visualizations (for more on the use of modern NLP methods for the study of word meaning, see [17]). To achieve this, we performed a text analysis via VOSviewer software (version 1.6.20) [18]. This software is able to analyze a significant amount of data and provide excellent network data mapping [19].

We analyzed the titles, abstracts, and keywords of the papers, and after preliminary cleaning (removing stopwords and broad terms like “conclusions”), we were able to reveal five clusters of relevant terms, known as co-occurrence maps, using the modularity-based clustering technique (Figure 2).

The terms are represented as nodes of varying sizes, proportional to the terms’ recorded frequency. Additionally, the analysis indicates the frequency with which the terms appear in close proximity to one another. 

These visualizations offer a high-level overview of the interconnectedness among items, helping us to identify the relevant themes and clusters within the dataset. Using this method, the following clusters were revealed:

1. **The red cluster**, which can be conditionally named as **“Nanomaterials and Agents”** for toothpaste production, is the largest (it consists of 24 items) and most central (i.e., related to all other clusters) one. It consists of the following terms: *bioactive glass nanoparticles (or bgn), bioglass, cement, calcium, calcium silicate, chitosan, csc, thickness, root canal sealer, nanogel*, etc. 

2. **The green**—second largest—cluster consists of 22 items. We name this cluster **“Materials and Methods for Regeneration of Bone and Teeth”**, and it consists of the terms *bone, hydrogel, implantation, infection, protamine, angiogenesis, amp, bmp, infection, osteogenesis, srphy, viscosity, zhphy. E faecalis (enterococcus faecali), and d301 group*.

3. **The yellow** cluster (10 items), named **“Materials for Mineralization of Hard Tooth”**, consists of the terms *acc, accpp, chororform, healing, migration, polyp fold eucalyptol, and hpdls*.

4. The central—red—cluster is closely related to the **purple** cluster **(10 items) “Antibiofilm Activity”**, which combines such terms as patient regeneration, periodontal regeneration, dental biofilm, dental pulp, dentin matrix, tissue regeneration, cariogenic condition, and molecular composition.

5. **The blue** cluster, which stands apart from the others (16 items) **“Agents and Methods for Prevention and Treatment of Dental/Oral Diseases”**, consists of the terms *enamel, medication, cariogenic bacterium, dental plaque, gapi, medication, control, propolis, sem, and surface roughness*.

Such a visualization gives the reader an overall understanding regarding the topics of this Special Issue and their interconnectedness.

We also analyzed the full texts of the papers using R package quanteda [20]. As a result, the most frequent words of the whole dataset of the articles were revealed and presented as a word cloud (Figure 3). This visualization method is an excellent option to help readers visually interpret a text and is useful for quickly gaining insight into the most prominent items in a given text by visualizing the word proportional to its frequency in a text collection (i.e., the larger the font size, the more frequent the word is throughout the whole Special Issue; we also used different colors for different font sizes for better visualisation).

We are confident that readers will find the contents of this Special Issue informative and engaging, and we extend our sincere gratitude to all the contributors who made this Special Issue possible. Thank you for your interest, and we hope you find it enlightening.

## Figures and Tables

**Figure 1:**
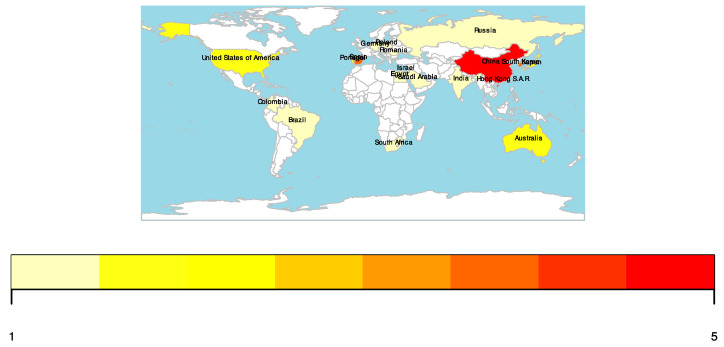
Geographic distribution of the corresponding authors (color bar indicates the number of articles from a given country).

**Figure 2:**
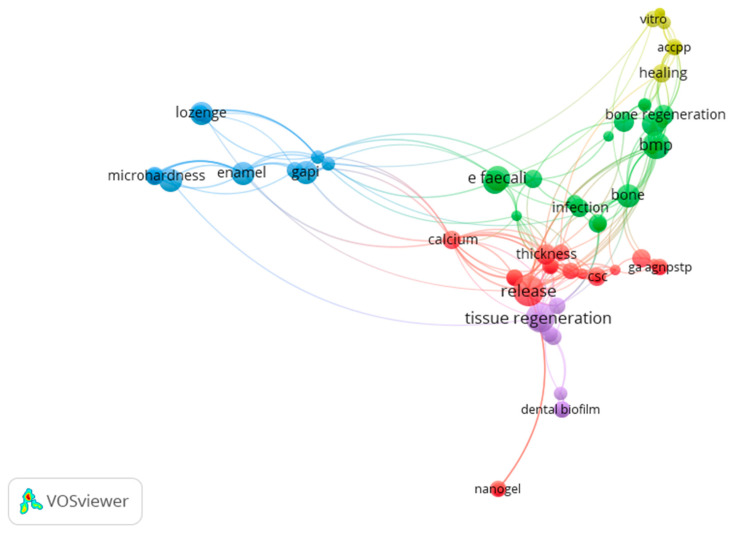
Clusters of terms.

**Figure 3:**
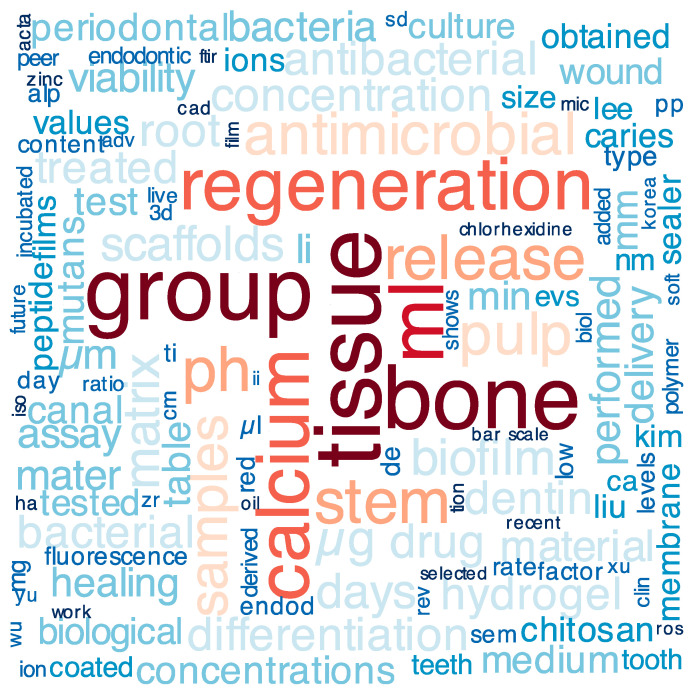
Word cloud with the most frequent words of the Special Issue.
